# A Fruitful Endeavor: Scent Cues and Echolocation Behavior Used by *Carollia castanea* to Find Fruit

**DOI:** 10.1093/iob/obaa007

**Published:** 2020-03-11

**Authors:** L B Leiser-Miller, Z A Kaliszewska, M E Lauterbur, Brianna Mann, J A Riffell, S E Santana

**Affiliations:** 1 Department of Biology, University of Washington, Seattle, WA 98195, USA; 2 Burke Museum of Natural History and Culture, University of Washington, Seattle, WA 98195, USA; 3 Department of Ecology and Evolutionary Biology, University of Arizona, Tucson, AZ 85721, USA

## Abstract

Frugivores have evolved sensory and behavioral adaptations that allow them to find ripe fruit effectively, but the relative importance of different senses in varying foraging scenarios is still poorly understood. Within Neotropical ecosystems, short-tailed fruit bats (*Carollia*: Phyllostomidae) are abundant nocturnal frugivores that rely primarily on *Piper* fruits as a food resource. Previous research has demonstrated that *Carollia* employs olfaction and echolocation to locate *Piper* fruit, but it is unknown how their sensory use and foraging decisions are influenced by the complex diversity of chemical cues that fruiting plants produce. Using free-ranging *C. castanea* and their preferred food, *Piper sancti-felicis*, we conducted behavioral experiments to test two main hypotheses: (1) foraging decisions in *C. castanea* are primarily driven by ripe fruit scent and secondarily by vegetation scent, and (2) *C. castanea* re-weights their sensory inputs to account for available environmental cues, with bats relying more heavily on echolocation in the absence of adequate scent cues. Our results suggest that *C. castanea* requires olfactory information and relies almost exclusively on ripe fruit scent to make foraging attempts. *Piper sancti-felicis* ripe fruit scent is chemically distinct from vegetation scent; it is dominated by 2-heptanol, which is absent from vegetation scent, and has a greater abundance of β-caryophyllene, β-ocimene, γ-elemene, and α-cubebene. Although variation in echolocation call parameters was independent of scent cue presence, bats emitted longer and more frequent echolocation calls in trials where fruit scent was absent. Altogether, these results highlight the adaptations and plasticity of the sensory system in neotropical fruit bats.

## Synopsis

Los frugívoros han evolucionado adaptaciones sensoriales y conductuales que les permiten encontrar frutos maduros de manera efectiva, pero aún existe una falta de conocimiento sobre la importancia relativa de los diferentes sentidos que los frugívoros utilizan en diferentes escenarios de forrajeo. Dentro de los ecosistemas neotropicales, los murciélagos de cola corta (*Carollia*: Phyllostomidae) son frugívoros nocturnos abundantes que dependen principalmente de los frutos de *Piper* como recurso alimentario. Previamente se ha demostrado que *Carollia* emplea el olfato y la ecolocalización para encontrar frutos de *Piper*, pero se desconoce cómo su uso sensorial y decisiones de forrajeo están influenciados por la compleja diversidad de señales químicas producidas por las plantas. En este trabajo, utilizamos *C. castanea* y su especie de frutos preferida, *Piper sancti-felicis*, para llevar a cabo experimentos conductuales y probar dos hipótesis principales: (1) las decisiones de forrajeo de *C. castanea* son incitadas principalmente por el aroma del fruto maduro y, en segundo lugar, por el aroma de la vegetación, y (2) *C. castanea* modula el uso de sus sentidos de acuerdo a las señales ambientales disponibles y utiliza la ecolocalización en mayor grado en ausencia de señales olfativas adecuadas. Nuestros resultados sugieren que *C. castanea* requiere información olfativa y se basa casi exclusivamente en el aroma del fruto maduro para hacer intentos de forrajeo. El aroma del fruto maduro de *P. sancti-felicis* es químicamente distinto al aroma de su vegetación; está dominado por 2-heptanol, el cual está ausente del aroma de la vegetación, y tiene una mayor abundancia de β-cariofileno, β-ocimeno, γ-elemeno, y α-cubibeno. Aunque encontramos que la variación en los parámetros de llamadas de ecolocalización es independiente de la presencia de diferentes señales aromáticas, los murciélagos emitieron llamadas de ecolocalización más largas y frecuentes en los experimentos donde el aroma de fruto estaba ausente. Conjuntamente, estos resultados destacan las adaptaciones y plasticidad del sistema sensorial de los murciélagos frugívoros neotropicales.

## Introduction

Animals rely on multiple sensory modalities to perform even the simplest ecological tasks (Burkhardt 1989; Siemers and Schnitzler 2000; Holland et al. 2005; Knaden and Graham 2016). One of the main goals of behavioral and sensory ecology lies in understanding the ability of different species to employ and modulate their sensory modes in the context of different environmental cues, and how the resulting behavioral decisions ultimately affect their ecology and evolution. Frugivores use various cues, including components of fruit color (Burkhardt 1989; Hiramatsu et al. 2008; Melin et al. 2008; Osorio and Vorobyev 2008; Valenta et al. 2013), shape (Von Helversen and Von Helversen 1999; Kalko and Condon 1998), and scent (Sánchez et al. 2006; Valenta et al. 2013) to find and select ripe fruit, and exhibit corresponding sensory specializations in their visual, auditory, and/or olfactory systems to target those cues (Catania 1999; Muller et al. 2007; Vanderelst et al. 2010). Little is known, however, about what scenarios facilitate or constrain sensory use and modulation during fruit location, selection, and acquisition in vertebrate frugivores. Insight into these processes would provide mechanistic understanding of the behaviors underlying their foraging ecology.

Frugivorous and omnivorous Neotropical leaf-nosed bats (Phyllostomidae) use three major senses, echolocation, olfaction, and vision, for navigation and foraging (e.g., Reiger and Jakob 1988; Gutierrez et al. 2018; Thiele and Winter 2005; Kalko and Condon 1998), which makes these organisms an exceptional system for investigating the relative role of different sensory modes during ecologically important tasks. Although there has been some work on phyllostomid vision, including comparisons of their short- and long-wave opsins (Müller et al. 2007, 2009; Kries et al. 2018; Sadier et al. 2018; Gutierrez et al. 2018), the importance of vision in ripe fruit location and selection has not been experimentally tested in phyllostomids. By contrast, experimental evidence strongly suggests that omnivorous phyllostomids rely primarily on echolocation to locate fruits (Kalko and Condon 1998), and specialized frugivores employ either olfaction or a combination of olfaction and echolocation to locate ripe fruit (Kalko and Schnitzler 1993; Korine and Kalko 2005; Hodgkison et al. 2007, 2013).

The relative importance of different sensory modes for fruit detection may depend on which plant cues can be readily perceived within a specific environmental context. To date, little is known about whether and how frugivorous phyllostomids integrate their primary sensory modes (echolocation and olfaction) conditional on which plant cues are present. While plant scents can travel over short or long distances (Riffell et al. 2014), they are rarely directional and may be difficult to detect in saturated scent environments such as rainforests. Conversely, echolocation allows for highly precise prey detection (Schnitzler and Kalko 2001; Brinkløv et al. 2009; Jakobsen et al. 2013), but phyllostomids emit short, high frequency, and low intensity calls (Thies et al. 1998; Korine and Kalko 2005). The echoes from these calls provide information about size, shape, texture, range, and position of an object in space relative to the bat (Simmons et al. 1975, 1983; Simmons and Stein 1980; Neuweiler 1989; Schmidt et al. 2000; Schnitzler and Kalko 2001), but these types of information are only effective at very short distances because low intensity, high frequency calls attenuate rapidly in warm, humid environments (e.g., 45–90 kHz attenuate at 1.4–4 dB/m at 25°C and 80% humidity; Jakobsen et al. 2013). Additionally, surrounding foliage can produce acoustic masking effects that may complicate fruit detection (Arlettaz et al. 2001; Korine and Kalko 2005). Therefore, flexibility in olfaction versus echolocation use could be highly beneficial for frugivorous bats given the limitations of each sensory mode within complex forest environments.

**Fig. 1:**
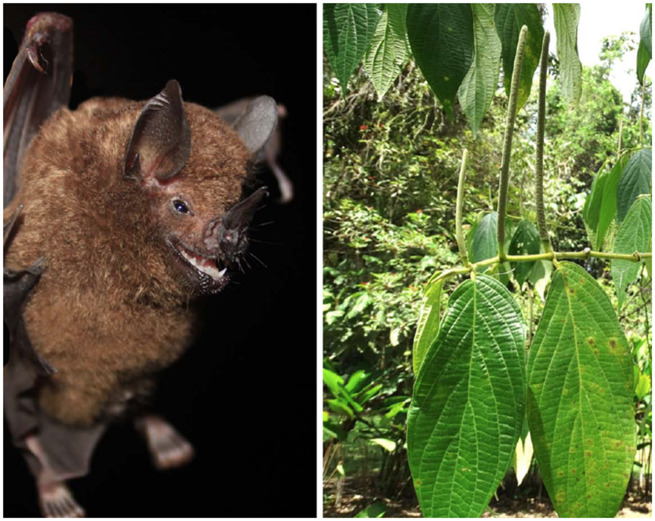
Study organisms, *Carollia castanea* (left) and *Piper sancti-felicis* (right). Photo credit: S.E. Santana.

Here, we study the primary plant cues from vegetation and fruits and how they relate to the roles of echolocation and olfaction for fruit detection and localization in the chestnut short-tailed fruit bat, *Carollia castanea*, a highly abundant frugivore and ecologically important seed disperser that inhabits forests in Central and South America (Bonaccorso 1979; Fleming 1991; Fig. 1). This research builds upon the seminal work of Thies et al. (1998), which demonstrated the importance of olfaction for fruit detection in *Carollia*; here we investigate the relative contributions of vegetative and fruit scent cues that drive *C.* *castanea*’s foraging decisions, and how varying foraging scenarios (i.e., changing of plant scent cues) may affect the relative reliance of these bats on olfaction versus echolocation for fruit detection. Evaluating the effect of vegetative scents on frugivore foraging behavior is particularly important because background scents from neighboring plants can impact the ability of animals to discriminate fruit or flower odors (Riffell et al. 2014) or, if vegetative scents are from the same plant, they can amplify the scent of the food source and make it more potent (e.g., Raguso and Willis 2005; Rusch et al. 2016; Stutz et al. 2016; McArthur et al. 2019).

*Carollia castanea* feeds primarily on infructescences of Neotropical *Piper* plants (Piperales: Piperaceae); the majority of fecal samples—in some cases 100%—from *C. castanea* collected in our study site contain *Piper* seeds (Lopez and Vaughan 2007; Maynard et al. 2019). *Piper* *sancti-felicis* (*P. scintillans*; Hammel et al. 2014) is the most abundant *Piper* species in *C. castanea*’s diet in this locality, and behavioral trials have demonstrated that these bats exhibit a preference for *P. sancti-felicis* over some other *Piper* species (Maynard et al. 2019). We test if foraging decisions in *C. castanea* are primarily driven by clear signals of food availability (i.e., *P. sancti-felicis* ripe fruit scent). We predict bats will cue in on ripe *P. sancti-felicis* fruit scent and secondarily on its vegetation scent, because the vegetation is a salient part of the plant that likely overlaps in its scent chemical composition with fruit scents, and thus a potential part of the olfactory cues used by *C. castanea* for fruit localization. Conversely, if vegetative scents differ sharply from those of ripe fruits, these could add sensory noise that may need to be filtered. Since these predictions rely on similarities of differences in the chemical composition of ripe fruit and vegetation scents, we conducted analyses contrasting the volatile chemical (scent) composition of these plant parts. Finally, we also hypothesize that *C. castanea* potentially re-weights their sensory inputs to account for available environmental cues, and predict that foraging bats emit echolocation calls more frequently when scent cues are absent. This is because *Carollia* can use echolocation for the final localization of fruit at close range, and perhaps also when searching for potentially edible fruit patches at a longer range (Thies et al. 1998; Corlett 2011). To test our hypotheses, we conducted a series of experiments to mimic the sensory challenges fruit bats may encounter in nature, and quantified differences in the bats’ behavioral responses when exposed to different sensory cues. Our study contributes to the understanding of which chemical cues bats use for fruit selection, which contexts facilitate alternating between sensory modes, and the behavioral and sensory adaptations phyllostomid fruit bats have evolved for foraging.

## Materials and methods

### Study animals

We used mist nets (Avinet, sizes: 4, 6, 9, and 12 m) to capture *C.* *castanea* along forest trails at La Selva Biological Station in Sarapiquí, Heredia Province, Costa Rica (Supplementary Table S1) from August to September 2016. All individuals were experimentally naïve and used in experiments only on the night of capture. Upon capture, each bat was kept in a clean cotton bag prior to experiments. We conducted experiments on 21 bats (16 adult males and 5 adult non-lactating, non-pregnant females), and if an individual had a positive trial, we subsequently collected biometric data and a 2–3 mm wing biopsy (Disposable Biopsy Punches, Integra Miltex) from its uropatagium. This was done for genetic analyses for a separate study and helped ensure we did not use recaptured individuals in subsequent experiments. All individuals were released near the site of capture after the behavioral experiments and processing were completed. All procedures were approved by the University of Washington Institutional Animal Care and Use Committee (Protocol No. 4307-02).

### Experimental set-up

**Fig. 2:**
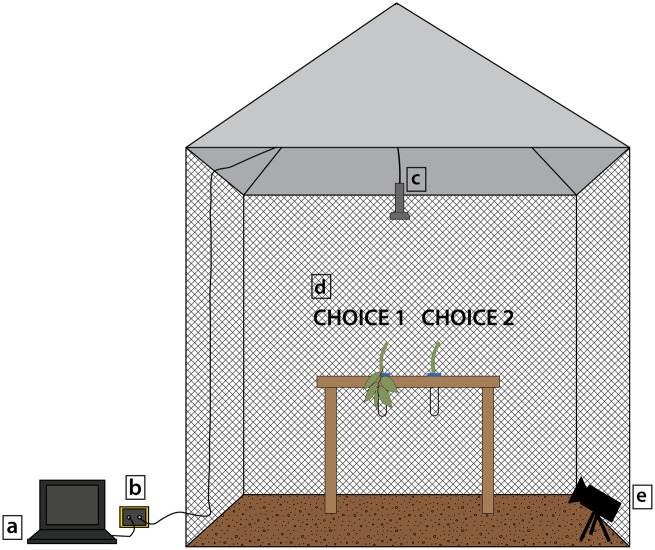
Diagram of experimental set-up: echolocation calls were visualized and recorded via a Dell 14 Rugged Extreme laptop (**a**) connected to a USG 116H recorder (**b**) that was connected to a CM16 condenser microphone (**c**). Target choice options were offered on a custom-made platform (**d**), here showing two example choice options, Choice 1: dummy with fruit scent and vegetation and Choice 2: dummy with fruit scent only. Bat behaviors were recorded with a Sony infrared-sensitive handycam (**e**).

We conducted two-choice behavioral experiments without reward inside a flight cage (Coleman, 3.048 × 3.048 × 2.1336 m) under natural ambient conditions at La Selva. As shown in Fig. 2, we placed an infrared-sensitive handycam (4K HD Video Recording, Sony, Japan) on a tripod at 30 cm height from the ground, which allowed us to record the bats’ foraging behaviors under infrared light conditions (>700 nm; beyond the spectral range of vision of phyllostomids; Jones et al. 2013). We recorded the bats’ echolocation calls with a condenser microphone (microphone capsule CM16, CMPA preamplifier unit, Avisoft Bioacoustics, Berlin, Germany) mounted at the top, center of the flight cage. During experiments, we visualized real-time calls using an ultrasound acquisition board (UltraSoudGate 116, Avisoft Bioacoustics; sampling rate 375 kHz, 16-bit resolution). At the back end of the flight cage, we placed a custom-built platform (90 cm long × 125 cm tall) which held two 50 mL falcon tubes, 40 cm apart, onto which we mounted each of the target (“fruit”) choices (Fig. 2). To control for size, shape, and material of these targets, we used three-dimensional (3D)-printed dummy fruits (Form 2 printer with FGLPWH02 resin) of the same shape and size of an average ripe *P.* *sancti-felicis* fruit. To mimic a ripe fruit, we smeared a dummy (3D printed) fruit with a standardized amount of ripe *P. sancti-felicis* fruit pulp (approx. 0.62 g, about one-third of a total fruit). We only used fruit collected on the same afternoon of each experimental trial night. We harvested vegetation (branches) from the same plants from which we collected ripe fruit. In trials with vegetation present, we placed the vegetation at the base of the dummy fruit, which is the natural configuration within the plant. Between each night of experiments, we cleaned dummy fruits with 95% ethanol to remove scents, rinsed them with water, and let them air dry at least 24 h before reusing.

**Fig. 3:**
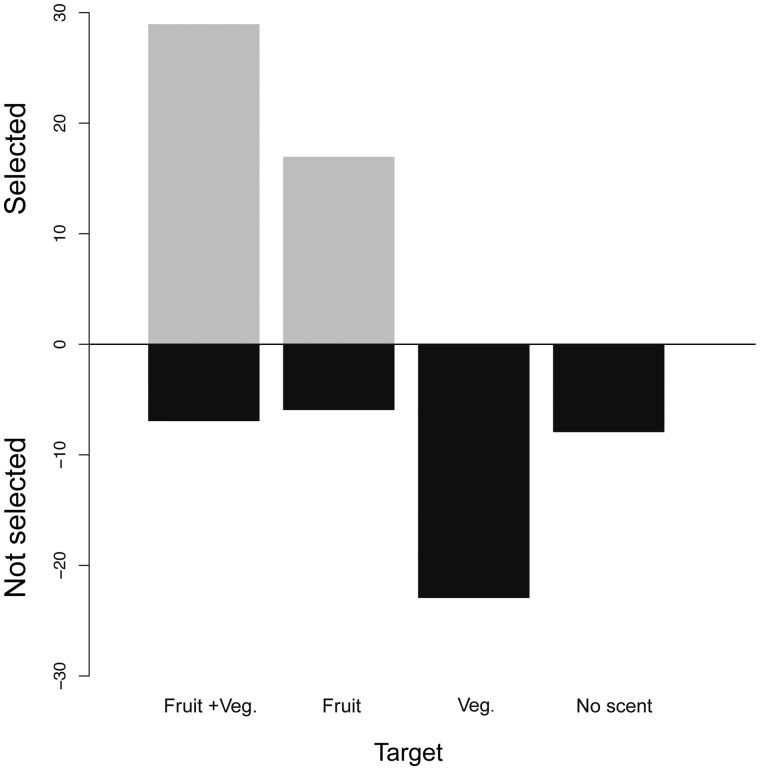
Summary of successful target selection by *Carollia castanea* for each of the four target types across behavioral experiments: dummy with fruit scent and vegetation (Fruit + Veg.), dummy with fruit scent only (Fruit), dummy unscented with vegetation only (Veg.), and dummy unscented (No scent).

To test our hypotheses, we presented each bat with a choice between two of the following targets during each experimental trial: 1) dummy with fruit scent (pulp from *P. sancti-felicis*), 2) dummy with vegetation only, 3) dummy with fruit scent and vegetation, and 4) dummy with no fruit scent or vegetation (Table 1). We ran each trial for a maximum of 20 min per bat, and subjected bats to up to four trials, conditional on their performance on the initial trial. If the bat did not perform within 20 min, we released the individual. Conversely, if the bat selected the fruit within the 20-min duration of the trial, we considered it a positive trial and began a new trial. To begin a new trial, one of us entered the flight cage and switched out choice targets. We randomized both the order we presented each trial and the position (left, right) of the target choice on the platform between consecutive trials to minimize confounding effects due to bat spatial learning (Thiele and Winter 2005). At the end of trials, we used a hand net to recapture the bat inside the flight cage, processed and released it, as described above.

**Table 1:** Description of the two target choices offered within each experimental treatment (T), and the response being tested during behavioral experiments on *Carollia castanea*

T	Choice 1	Choice 2	Test	Number of trials
1	Dummy unscented + vegetation	Dummy with fruit scent + vegetation	Preference for fruit scent in the presence of vegetation scent	36
2	Dummy unscented + vegetation	Dummy with fruit scent	Preference between vegetation and fruit scents	23
3	Dummy with fruit scent + vegetation	Dummy with fruit scent	Preference for vegetation scent in the presence of fruit scent	23
4	Dummy unscented	Dummy unscented + vegetation	Preference for vegetation scent	8

Number of trials differed among treatments due to differences in the number of positive responses of experimental bats (see the “Materials and Methods” section).

### Analysis of flight behavior during target search and approach

We watched videos of the behavioral trials at normal playback speed on a computer at the University of Washington, Seattle, WA, USA. From each video, we recorded: the amount of time it took the bat to select one of the target choices presented, the real time of making a selection (to synchronize with acoustic calls), and the individual’s choice. We defined a target selection event as a bat landing on a target and attempting to bite it. We noted additional characteristics of the bat’s flight behavior (e.g., exploratory flights around the flight cage) and target exploration (e.g., approaching or hovering over target) for all trial videos.

### Analysis of echolocation behavior during target approach

We analyzed echolocation calls emitted during target approach using Avisoft SASLabPro v. 4.40 (Avisoft Bioacoustics). Target approach did not always result in target selection, as defined above, in which case we considered it as a target exploration behavior. We used the time of target exploration and selection events from video recordings and matched them with the time stamps of the call files to synchronize acoustics with the recorded events. These files were used in the subsequent analyses, and included the acoustic calls for 1 min prior to target exploration and selection events, which we defined as the approach window. We chose a 1-min interval prior to these events because we were not only interested in sensory behaviors for target localization during selection (typically seen in the approach phase), but also in the sensory behavior when “searching” for food. *Carollia castanea*, similar to other phyllostomids, emits calls well >20 kHz (Thies et al. 1998; Brinkløv et al. 2011), so we used this as the cut-off frequency to avoid including noise from recording at high gain in ambient conditions (Geipel et al. 2013). We filtered each acoustic sequence using a high-pass filter (at 20 kHz) and visualized spectrograms using a Hamming window (512 fast Fourier transform, 98.95% overlap). We extracted the following echolocation call parameters for comparisons across trial types: maximum frequency (kHz), minimum frequency (kHz), peak frequency (i.e., frequency with the highest amplitude, kHz), call duration (ms), call interval (ms), and total bandwidth (kHz) from the spectrograms at the maximum energy of each call. We compiled sequences per individual (approximately 8–20) and calculated mean and standard deviation for each call parameter per trial type per individual (Table 2).

**Table 2:** Means (±standard deviation) of echolocation call parameters for *Carollia castanea* during each experimental treatment (T, from Table 1)

T	Duration (ms)	Interval (ms)	Peak frequency (kHz)	Minimum frequency (kHz)	Maximum frequency (kHz)	Bandwidth (kHz)
1	2.09 ± 1.02	314.7 ± 252.8	83.9 ± 3.44	62.5 ± 10.0	108.3 ± 9.131	45.8 ± 15.0
2	1.34 ± 0.0103	268.1 ± 151.5	85.9 ± 6.89	68.0 ± 9.79	111.7 ± 12.54	43.7 ± 19.1
3	1.70 ± 0.0589	421.8 ± 20.63	83.1 ± 5.69	60.7 ± 6.16	111.3 ± 4.531	50.6 ± 4.92
4	2.71 ± 0.206	81.6 ± 26.8	87.2 ± 10.65	64.7 ± 10.7	108.9 ± 11.51	44.2 ± 11.6

Means for the call parameters were calculated by averaging the calls for each individual bat across its entire calls sequence (the approach call for one individual, for one treatment), and averaging each of these values across individuals within each treatment.

### Statistical analyses of behavioral experiments

We performed all statistical analyses in R v. 3.2.4 (R Core Team 2019). We used Chi-squared tests to assess the differences in bat preference among target types. We tested the normality of the echolocation call data using Shapiro–Wilks tests (Shapiro and Wilk 1965) and subsequently log-transformed these data to improve normality. We compared the differences in call parameters among trial types using analysis of variance (ANOVA).

### Target headspace comparisons

To determine if *P. sancti-felicis* ripe fruit and vegetation present similar or different olfactory cues to *C. castanea*, we compared the volatile organic compounds (VOCs) that make up the scents of these plant parts. We collected vegetation (6.7–18.1 g of fresh weight leaf material; one branch; *n* = 12) and ripe fruit (6.0–41.0 g, fresh weight; 2–35 fruits; *n* = 9) samples from *P. sancti-felicis* plants at La Selva. A relatively large sample of ripe fruit was necessary for VOC capture and detectability by our experimental setup. We collected VOCs from these samples via dynamic headspace adsorption using a push–pull system (Raguso and Pellmyr 1998; Riffell et al. 2008). Within 2 h of collection, we placed each sample in a 3 L nylon bag (Reynolds, Richmond, VA, USA) and connected the bag to a diaphragm pump (400-1901, Barnant Co., Barrington, IL, USA) that pulled the fragrant headspace air through a sorbent cartridge trap (50 mg Porapak Q with silanized glass wool Waters Corp., Milford, MA, USA) and pushed air though a charcoal filter. We collected VOCs in this manner for 20 h per sample, following previously established protocols and to ensure characterization of the full chemical profile (Byers et al. 2014). We eluted trapped volatiles from each sample’s sorbent cartridge with 600 μL of HPLC-grade hexane into a 2 mL borosilicate glass vial with a Teflon-lined cap. Subsequently, we stored all of the samples at –20 to –80°C. We analyzed a 3 μL aliquot of each sample using an Agilent 7890A Gas Chromatograph (GC) and a 5975C Network Mass Selective Detector (Agilent Technologies, Palo Alto, CA, USA). To separate the VOCs, we used a DB-5MS GC column (J&W Scientific, Folsom, CA, USA; 30 m, 0.25 mm, and 0.25 μm) with helium as the carrier gas flowing at a constant rate of 1 cc per min (Byers et al. 2014). The initial oven temperature was 45°C for 4 min, followed by a heating gradient of 10°C per min to 230°C, which was then held isothermally for 4 min. We initially identified the chromatogram peaks with the aid of NIST 08 mass spectral library (v. 2.0f; ca. 220,460 spectra of 192,108 different chemical compounds) followed by verification using alkane standards and comparing with published Kovats indices. Standards of β-caryophyllene, germacrene D, and β-pinene (>98% purity; Sigma–Aldrich, St. Louis, MO, USA) were run to verify peak identities. We integrated the peaks for each compound using ChemStation software (Agilent Technologies) and present them in Table 4.

**Table 4:** Abundance of volatile organic compounds (VOCs) found in the scent of *Piper sancti-felicis* ripe fruit and vegetation, sorted according to their Kovats retention indices (KRI)

Chemical name	IUPAC	KRI	Class	Fruit mean	Fruit*N* present	Fruit mean(*N* present)	Veg. mean	Veg.*N* present	Veg. mean(*N* present)
3-Hexene-1-ol	hex-3-en-1-ol	858	Aliphatic alcohol	0	0	0	0.143	8	0.214
2-Heptanol	Heptan-2-ol	881	Aliphatic alcohol	0.128	3	0.384	0	0	0
Ethyl tiglate	Ethyl-2-methylbut-2-enoate	952	Fatty acid ester	0	0	0	0.045	3	0.181
3-Hexen-1-ol, acetate	[hex-3-enyl] acetate	1001	Acetate ester	0	0	0	0.009	3	0.037
β-Pinene	6,6-Dimethyl-4-methylidenebicyclo[3.1.1]heptane	1003	Monoterpene	0.149	5	0.268	0.114	11	0.125
3-Carene	4,7,7-Trimethylbicyclo[4.1.0]hept-3-ene	1010	Monoterpene	0.003	1	0.024	0.004	3	0.017
α-Terpinene	1-Methyl-4-propan-2-ylcyclohexa-1,3-diene	1020	Monoterpene	0.016	4	0.036	0.060	8	0.090
*p*-Cymene	1-Methyl-4-propan-2-ylbenzene	1028	Aromatic	0.080	5	0.145	0.072	8	0.108
β-Ocimene	3,7-Dimethylocta-1,3,6-triene	1050	Monoterpene	0.111	6	0.167	0.003	3	0.012
γ-Terpinene	1-Methyl-4-propan-2-ylcyclohexa-1,4-diene	1064	Monoterpene	0.022	5	0.039	0.052	8	0.078
Terpinolene	1-Methyl-4-propan-2-ylidenecyclohexene	1091	Monoterpene	0.001	1	0.005	0.026	8	0.040
Ethyl benzoate	Ethyl benzoate	1175	Aromatic	0.044	4	0.100	0.083	10	0.099
α-Cubebene	4,10-Dimethyl-7-(propan-2-yl)tricyclo[4.4.0.0^1,5^]dec-3-ene	1354	Sesquiterpene	0.067	2	0.300	0.005	4	0.014
β-Elemene	2,4-Diisopropenyl-1-methyl-1-vinylcyclohexane	1394	Sesquiterpene	0.027	3	0.080	0.071	9	0.094
γ-Elemene	1-Ethenyl-1-methyl-4-propan-2-ylidene-2-prop-1-en-2-ylcyclohexane	1429	Sesquiterpene	0.091	3	0.273	0.041	3	0.163
β-Caryophyllene	4,11,11-Trimethyl-8-methylidenebicyclo[7.2.0]undec-4-ene	1433	Sesquiterpene	0.169	8	0.190	0.080	11	0.088
α-Bergamotene	4,6-Dimethyl-6-(4-methylpent-3-enyl)bicyclo[3.1.1]hept-3-ene	1440	Sesquiterpene	0.005	2	0.021	0.027	5	0.064
α-Caryophyllene	2,6,6,9-Tetramethylcycloundeca-1,4,8-triene	1470	Sesquiterpene	0.005	2	0.021	0.004	2	0.021
γ-Muurolene	7-Methyl-4-methylidene-1-propan-2-yl-2,3,4a,5,6,8a-hexahydro-1H-naphthalene	1485	Sesquiterpene	0.003	2	0.011	0.019	6	0.037
Germacrene D	1-Methyl-5-methylidene-8-propan-2-ylcyclodeca-1,6-diene	1495	Sesquiterpene	0.034	3	0.103	0.059	9	0.079
Bicyclogermacrene	3,7,11,11-Tetramethylbicyclo[8.1.0]undeca-2,6-diene		Sesquiterpene	0.035	1	0.319	0.018	3	0.073
Alloaromadendren	1,1,7-Trimethyl-4-methylidene-2,3,4a,5,6,7,7a,7b-octahydro-1aH-cyclopropa[e]azulene	1504	Sesquiterpene	0.009	3	0.027	0	0	0
α-Bulnesene	3,8-Dimethyl-5-prop-1-en-2-yl-1,2,3,3a,4,5,6,7-octahydroazulene	1505	Sesquiterpene	0	0	0	0.038	3	0.151

International Union of Pure and Applied Chemistry (IUPAC) nomenclature name is provided for each VOC. Ripe fruit mean and vegetation (Veg.) mean values are the mean proportions across all samples (*n* = 9 fruit; *n* = 12 vegetation); *N* present is the number of samples in which the VOC was found in ripe fruit and vegetation, respectively; fruit or veg. mean (*N* present) are the mean proportions calculated only across the samples in which the VOC was found in ripe fruit and vegetation (i.e., *N* present), respectively.

## Results

### Target preference

Most individuals performed exploratory flights prior to showing interest in the presented target choices. These behaviors consisted of circling flights around the cage without approaching the target. In most trials (83%), bats attempted to remove a target by landing and trying to bite the dummy fruits (Supplementary Video S1). Comparisons across treatments revealed that *C. castanea* strongly preferred targets consisting of a dummy fruit with fruit scent (*n* = 10, *χ*^2^ = 6.21, *P* = 5.69e−05) or a dummy fruit with fruit scent and vegetation (*n* = 13, *χ*^2^ = 22.154, *P* = 2.52e−06) over targets that had an unscented dummy fruit and vegetation (Fig. 3). The presence of vegetation did not affect the bats’ preferences for fruit scent (dummy fruit with fruit scent versus dummy fruit with fruit scent and vegetation: *n* = 13, *χ*^2^ = 0, *P* = 0.99). Bats never chose unscented dummy fruits, either alone or with vegetation.

### Echolocation behavior

**Fig. 4:**
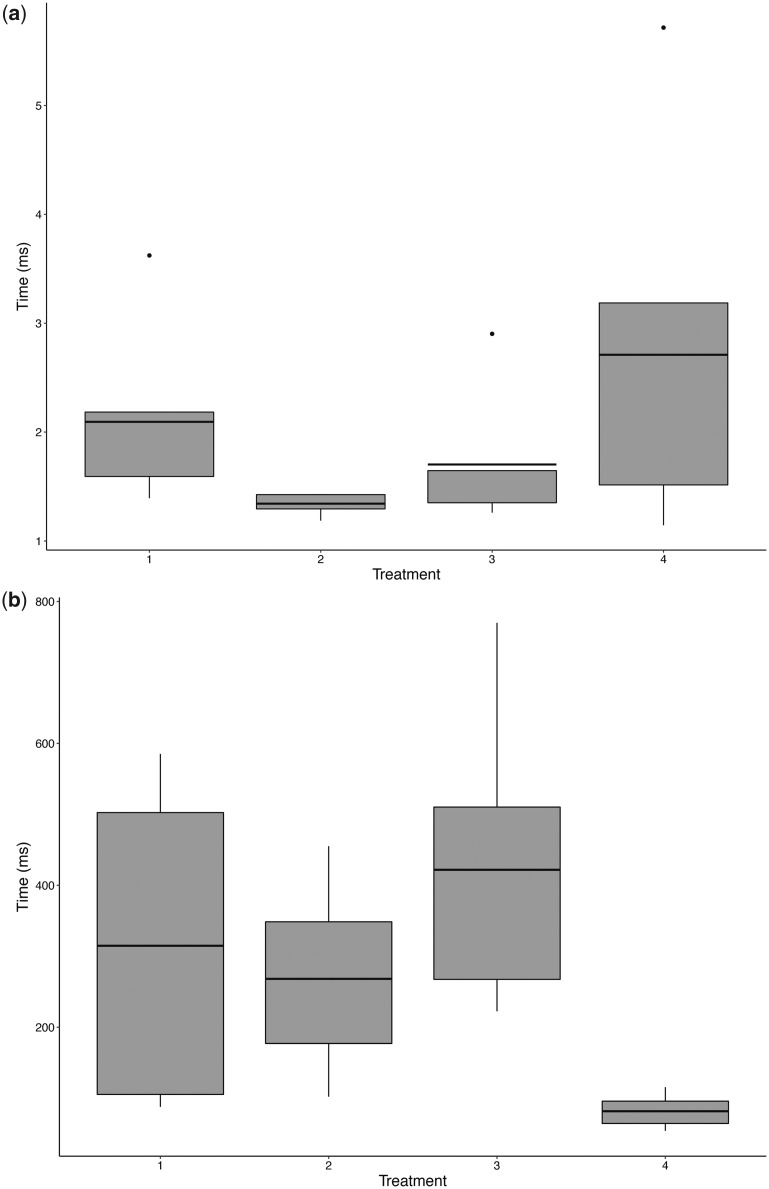
Summary of the duration of *Carollia castanea*’s echolocation calls across treatments (**a**) and summary of the interval of *C. castanea*’s echolocation calls across treatments (**b**). Treatments are described in Table 1.

All bats used in the experiments emitted echolocation calls throughout the trials. We did not find statistically significant differences in the echolocation call parameters among treatment types during the approach window (minimum frequency, maximum frequency, peak frequency, bandwidth, duration, pulse interval, all *P* > 0.05; Table 3). However, there were marked trends in call duration and interval in some trial types. Bats emitted echolocation calls more frequently (shorter interval) and of longer duration in treatments where no fruit scent was present (unscented dummy vs. unscented dummy with vegetation; Fig. 4).

**Table 3:** Summary of ANOVA results comparing each call parameter trait across the four experimental treatments (from Table 1)

Variable	DF	Sum. Sq.	Mean Sq.	*F*-value	*P*
Duration	1	0.852	0.852	0.728	0.405
Interval	1	1.37	1.37	1.209	0.287
Peak frequency	1	0.36	0.361	0.194	0.665
Minimum frequency	1	0.00	0.0001	0.00	0.995
Maximum frequency	1	0.00	0.002	0.001	0.975
Bandwidth	1	0.00	0.0004	0.000	0.989

### Chemical differences between targets

The scent profiles of *P. sancti-felicis* vegetation and ripe fruit differ slightly in VOC composition, and greatly in the proportion of specific VOCs (Table 4). The vegetation scent of *P. sancti-felicis* is dominated by 3-hexene-1-ol, α-bulnesene, and ethyl tiglate, which are not found in the ripe fruit scent. The ripe fruit scent of *P. sancti-felicis* is dominated by 2-heptanol, which is not found in vegetation scent, and is also characterized by a greater abundance of β-caryophyllene, β-ocimene, γ-elemene, and *α*-cubebene. Both vegetation and fruit scents have a high abundance of *p*-cymene and β-pinene (Table 4).

## Discussion

An animal’s sensory ecology and behavior is shaped by the environment it inhabits, as well as its evolutionary history. As such, certain sensory modalities play key roles in mediating ecological interactions. Mammalian frugivores are able to locate and acquire ripe fruit by using and integrating across sensory modalities: they use vision to detect differences in fruit color and luminance (Burkhardt 1989; Hiramatsu et al. 2008; Osorio and Vorobyev 2008; Valenta et al. 2013; Melin et al. 2017, 2019), olfaction to detect individual VOCs or entire odor plumes (Sánchez et al. 2006; Valenta et al. 2013) and, in the case of phyllostomid bats, echolocation to gather information about fruit shape and location (Kalko and Condon 1998; Von Helversen and Von Helversen 1999). Our results indicate that *C. castanea* makes foraging decisions based on ripe fruit scent over all other cues presented, but may rely more heavily on echolocation when adequate olfactory cues are absent. Importantly, in the absence of plant scent cues, a *P. sancti-felicis* fruit shape does not elicit a target selection response from *C. castanea*. This gives further support to the primary role of olfaction, followed by echolocation, when these bats forage for fruit.

The importance of olfaction for foraging in *C. castanea* is supported by previous research on other *Carollia* species that rely less on *Piper* as a food resource. For example, *C. perspicillata* can recognize minute concentrations of particular chemical components (fruit-typical odor components like ethyl butyrate, *n*-pentyl acetate, or linalool; Laska 1990), are attracted to fruit scent even when no other cues are present (Hessel and Schmidt 1994), and visit mist nets spiked with the essential oil of *Piper gaudichaudianum* more frequently (Mikich et al. 2003). Here, we link the bouquet of VOCs from a known, preferred food source with the behavioral preferences of *C. castanea*. Our experimental results strongly suggest that *C. castanea* uses ripe fruit scent, as opposed to a combination of ripe fruit and/or vegetation scent, or fruit shape, as the cue to locate food items. Our chemical analyses of ripe fruit and vegetation VOCs provide an explanation for this pattern: the scent profile of the *P. sancti-felicis* ripe fruit and vegetation is somewhat similar in composition, but differ greatly in abundance of some specific VOCs. Additionally, the ripe fruit scent profile contained a few distinct VOCs that were not found in vegetation scent, and vice versa. Considering that *C. castanea* forages in a complex sensory environment, the forest understory, it may be advantageous for the bats to cue in specific chemicals that unmistakably signal fruit ripeness against a background of unripe fruit and vegetation within a *Piper* bush, as well as adjacent vegetation. Our results motivate future work to examine whether and how some of these key volatiles—or their combinations—may signal fruit ripeness among the vegetative mélange.

Echolocation calls did not differ significantly in frequency between trial types, suggesting that *C. castanea* has a stereotyped call structure regardless of their foraging tasks. While this has not been broadly studied, having a stereotyped echolocation call is common in phyllostomids (Kalko and Condon 1998; Thies et al. 1998; Korine and Kalko 2005; Geipel et al. 2013). Nevertheless, our experiments revealed that *C. castanea* potentially modulates time-linked echolocation traits (i.e., duration of the call and time between calls, interval) when confronted with different food cues. As in all mammals, phyllostomid bats process olfactory cues by inhaling air through their nose, and also emit echolocation calls out of their nose. Because of the potential conflict posed by performing these two functions simultaneously by the nasal cavity, we propose that frugivorous phyllostomids exhibit behavioral modulation in their nasally-linked senses to alternate between—and maximize the effectiveness of—one sensory cue versus another, when appropriate.

Echolocation provides bats with high-resolution information about shape, surface texture, and material of an object at close range (Schnitzler et al. 1983; Ostwald et al. 1988; Kober and Schnitzler 1990), but bats also use echolocation for navigation and detection of plants that signal through morphology for better acoustic detection. We saw a general trend of longer duration of echolocation calls and shorter intervals between calls when bats were offered choices that did not include a ripe fruit scent cue. If ripe fruit scent is the primary cue for fruit location and selection by *C. castanea*, why are there differences in echolocation call duration and interval between treatments with and without ripe fruit scent? Decreased time between calls (interval) and longer duration means these bats were calling more frequently in the absence of ripe fruit scent. We hypothesize that, when ripe fruit odor cues are absent, *C. castanea* relies more heavily on echolocation to locate a potential food item, in this case one that may resemble an edible *Piper* fruit. In contrast, when bats were presented with any target that had ripe fruit scent (one or two choices), they emitted shorter echolocation calls at longer intervals, thus echolocating less frequently. We hypothesize that this decrease in echolocation call duration and increase in interval could be linked to an increase in the bats’ use of olfaction as they attempt to locate edible ripe fruits or determine which one is the “most edible” option.

Furthermore, previous studies have demonstrated that phyllostomid bats can use echolocation to pinpoint the position of a fruit (Kalko and Condon 1998), and echolocating bat species, in general, alter call parameters to overcome acoustic masking effects during prey location (Kalko and Schnitzler 1993). This can be accomplished by changing the duration and interval of the call (Siemers and Schnitzler 2000); bats typically extend the duration of a call when searching for prey and during orientation flights (Kalko and Schnitzler 1993), and decrease the time between calls when approaching a prey item (Kalko and Schnitzler 1993; Siemers and Schnitzler 2000). In our experiments, bats never chose unscented dummy fruits as potential food options, but our behavioral and acoustic recordings demonstrated that they did probe them via echolocation. We propose that *C. castanea* has a series of criteria (e.g., fruit scent, shape, configuration of fruit in relation to vegetation), which may be hierarchical, and are integrated during the search and localization of a potential food item.

The use of echolocation and olfaction for food selection has been documented in other frugivorous and omnivorous phyllostomids. *Artibeus jamaicensis* is a specialized frugivore that detects, localizes, and classifies ripe fruits primarily by olfaction (Kalko et al. 1996). In contrast, *Phyllostomus hastatus*, a large omnivorous bat, consistently uses echolocation over olfaction when foraging for *Gurania spinulosa*, a pendulous fruit-bearing vine (Kalko and Condon 1998). These examples illustrate an echolocation–olfaction continuum across phyllostomids that forage for fruit, and suggest that multiple sensory modes are important for fruit foraging in complex environments. They also substantiate that sensing mode could be conditional on which food cues are present or the degree of specialization of each species (e.g., omnivores versus specialized frugivores). To date, it is still unclear which senses are most important for fruit foraging in most bat frugivores, and what facilitates the use of one sense over another.

This study provides behavioral links between a frugivore’s sensory abilities and plant chemical cues, a relationship that is critical to understanding the ecological dynamics and coevolution between plants and their seed dispersers. There is still much to learn about how vertebrate frugivores perceive and interact with their potential food sources, thus further observational and experimental studies are critical for determining what specific fruit traits (e.g., compounds or combination of compounds in ripe fruit) drive fruit selection by frugivorous species.

## Supplementary Material

obaa007_Supplementary_Data
