# Supplementary material for: A Fruitful Endeavor: Scent Cues and Echolocation Behavior Used by Carollia castanea to Find Fruit
Source: Integr Org Biol. 2020 Mar 11;2(1):obaa007. doi: 10.1093/iob/obaa007 (PMC7671165; doi:10.1093/iob/obaa007)
Supplement: obaa007_Supplementary_Data [file obaa007_supplementary_data.zip › Supplementary Table 1 2019-038.docx]

**Supplementary Table 1:** List of individual bats used in successful experiments and associated biometric data: M (male), F (female), A (adult), NP-NL (not pregnant, not lactating), NS (non-scrotal).

| No. | Species | ID | Sex | Age | Rep. Cond. | Mass (g) | FA (mm) |
| --- | --- | --- | --- | --- | --- | --- | --- |
| 1 | *Carollia castanea* | 083016_1 | F | A | NP-NL | 14 | 37.3 |
| 2 | *Carollia castanea* | 083016_2 | M | A | NS | 12 | 35.8 |
| 3 | *Carollia castanea* | 083016_3 | M | A | NS | 11 | 37.4 |
| 4 | *Carollia castanea* | 083116_1 | M | A | NS | 14 | 37.9 |
| 5 | *Carollia castanea* | 083116_2 | M | A | NS | 12 | 36.1 |
| 6 | *Carollia castanea* | 083116_3 | F | A | NP-NL | 14 | 37.1 |
| 7 | *Carollia castanea* | 090116_2 | M | A | NS | 13 | 36.8 |
| 8 | *Carollia castanea* | 090116_3 | M | A | NS | 14 | 37.6 |
| 9 | *Carollia castanea* | 090816_2c | M | A | NS | 14 | 36.7 |
| 10 | *Carollia castanea* | 090816_3c | M | A | NS | 13 | 36.3 |
| 11 | *Carollia castanea* | 090816_4c | F | A | NP-NL | 14 | 36.5 |
| 12 | *Carollia castanea* | 090816_5c | M | A | NS | 11 | 36 |
| 13 | *Carollia castanea* | 090816_6c | M | A | NS | 11 | 36.2 |
| 14 | *Carollia castanea* | 090916_1 | F | A | NP-NL | 14 | 38.7 |
| 15 | *Carollia castanea* | 090916_2 | M | A | NS | 14 | 37.1 |
| 16 | *Carollia castanea* | 090916_3 | M | A | NS | 11 | 37.2 |
| 17 | *Carollia castanea* | 090916_5 | M | A | NS | 12 | 36.2 |
| 18 | *Carollia castanea* | 091016_1 | F | A | NP-NL | 12 | 36.2 |
| 19 | *Carollia castanea* | 091016_2 | M | A | NS | 13 | 37.2 |
| 20 | *Carollia castanea* | 091016_4 | M | A | NS | 14 | 37.1 |
| 21 | *Carollia castanea* | 091216_3 | M | A | NS | 10 | 36.2 |
